# The research hotspots and trends of artificial intelligence technology in nursing management: a bibliometric study

**DOI:** 10.3389/fmed.2025.1710269

**Published:** 2025-12-10

**Authors:** Jianhua Li, Lu Zhang, Qing Hou, Shanshan Jiang, Lanfang Shen, Junfan Wei

**Affiliations:** 1 Department of Nursing, Nanjing Central Hospital, Nanjing, China; 2 Department of Emergency, Nanjing Central Hospital, Nanjing, China; 3 Department of Management Office of Hexi Outpatient, Nanjing Central Hospital, Nanjing, China

**Keywords:** artificial intelligence, nursing management, bibliometric analysis, machine learning, digital health, research trends, health informatics

## Abstract

**Background:**

Artificial intelligence (AI) has emerged as a transformative force in healthcare, with nursing management being a key area of application. As AI technologies such as machine learning and decision support systems are increasingly integrated into clinical workflows, understanding the research landscape of AI in nursing management becomes essential.

**Methods:**

A total of 151 English-language publications from the Web of Science Core Collection and Scopus (data from 1990 to August 2025) were analyzed using CiteSpace, VOSviewer, and Bibliometrix. Analyses included co-authorship networks, keyword co-occurrence, citation patterns, and trend visualizations.

**Results:**

Since 2017, the number of relevant publications has surged, with China leading in output and the United States leading in collaborative centrality. Key institutions include Columbia University and Capital Medical University. Collaboration among authors remains limited, though several researchers exert significant influence. Five major research clusters have been identified, covering decision support, nursing leadership, informatics, behavioral aspects, and disease-specific applications. Emerging hotspots include “nursing management,” “algorithms,” and “deep learning.”

**Conclusion:**

In the field of nursing management, AI is transitioning from conceptual to practical application, demonstrating significant potential for enhancing decision-making and improving patient care. However, the field remains fragmented, with limited collaboration among authors and institutions. This study highlights AI’s potential to transform nursing management while emphasizing the need for closer interdisciplinary and international cooperation. Future research should focus on addressing ethical concerns such as data privacy and transparency, and developing AI tools that integrate more effectively into nursing practice. While this study offers valuable insights, there are limitations, including the exclusion of non-English literature and reliance on bibliometric analysis, which may not fully reflect AI’s real-world clinical applications. Looking ahead, fostering collaboration, improving ethical governance, and optimizing AI tools will be key to advancing AI in nursing management.

## 1 Introduction

Over the past few decades, artificial intelligence (AI) has evolved from a theoretical concept into a transformative power across multiple industries, with healthcare emerging as one of its most influential fields (1). AI refers to a domain of computer science dedicated to developing systems capable of executing tasks typically associated with human intelligence. These tasks include learning, inference, solving problems, interpreting oral language, perception, and even creativity (2). Advancements in AI—driven by technologies such as machine learning, deep learning, natural language processing, computer vision, and others—have enabled the development of intelligent tools that assist healthcare professionals in carrying out complex tasks, including data interpretation, clinical decision-making, risk assessment, and workflow coordination, thereby enhancing the quality and efficiency of healthcare management (3, 4). These capabilities not only revolutionize clinical diagnosis and treatment but also extend into administrative and management fields within healthcare systems, leading to new possibilities for innovation (5).

One of the most promising fields for AI application is nursing management, which serves as a core pillar of healthcare delivery, involving the organization, oversight, and optimization of nursing staff, processes, and patient care outcomes (6). Nursing management plays a critical role in ensuring the efficiency, quality, and safety of patient care (7). It includes a wide range of responsibilities, such as staff recruitment and scheduling, developing patient care plans, evaluating performance, ensuring quality assurance, allocating resources, and complying with healthcare policies (8). Beyond daily operations, nursing managers are also expected to lead strategic initiatives like staff development, revolution management, and digital technology implementation (9). In this study, “nursing management” is operationally defined as the systematic planning, organization, coordination, and evaluation of nursing resources, workflows, and personnel to ensure the quality, safety, and efficiency of patient care. It differs from general nursing or clinical care in that it primarily focuses on administrative and leadership functions—such as human resource allocation, quality improvement, decision support, and organizational governance—rather than direct patient care delivery. As the global healthcare system continues to grapple with workforce shortages, aging populations, and increasing care demands, the need for data-driven, intelligent, and adaptive strategies in nursing management continues to rise (10).

In recent years, AI applications in real-world settings have demonstrated significant value in enhancing the efficiency and effectiveness of nursing management practices. In this context, “AI technology” in the context of this research refers to computational systems and algorithms capable of simulating cognitive processes such as learning, reasoning, and decision-making to assist managerial and clinical operations in nursing. The scope of AI technologies considered in this study encompasses machine learning, deep learning, natural language processing, computer vision, robotic process automation, and decision-support systems applied within nursing administration and management workflows. For instance, AI-powered patient monitoring systems have been deployed to detect early signs of deterioration, predict falls, and utilize facial recognition tools to assess patient distress. These technologies provide actionable insights to nursing managers, enabling improvements in care quality and safety (11). These technologies not only enhance bedside care but also reduce nursing workload, enabling optimal allocation of human resources and thereby improving the operational capacity of healthcare teams (12). In hospital management, AI-driven communication platforms have been successfully integrated into perioperative workflows to automate patient engagement, collect preoperative data, and reduce surgical cancelations (6). Additionally, AI virtual assistants have been deployed for routine patient consultations, real-time triage, and multilingual communication support, thereby expanding the reach of care services without increasing the workload on staff (13). In terms of management, intelligent human resource systems—such as AI-assisted scheduling, performance tracking, and workload forecasting—are increasingly assisting nursing leaders in strategic decision-making and resource planning (6). The emergence of these studies reflects a growing recognition that AI can serve as a reliable assistant in nursing management, enabling evidence-based, data-driven management to support staff wellbeing and patient outcomes (11).

Despite the rapid expansion of the literary body in this field, most research remains fragmented. Significant variations in research focus, contextual frameworks, and methodological rigor often make it challenging to develop a coherent understanding of the field’s evolution. Furthermore, the absence of a unified understanding of thematic hotspots, disciplinary frontiers, and collaborative networks hinders the identification of research gaps and strategic priorities. While there were several reviews (14, 15) on AI in healthcare and nursing have primarily adopted narrative or scoping methodologies focusing on clinical applications, ethical concerns (16), or implementation barriers, this study fills a distinct gap by offering a structural and network-based analysis of the field. Specifically, our bibliometric approach systematically maps institutional collaboration patterns, research productivity by country, co-authorship networks, and keywords analysis—dimensions that are often overlooked in traditional reviews. By revealing fragmented collaborative structures and identifying underrepresented research clusters, this study not only quantifies global trends but also highlights strategic gaps in interdisciplinary convergence and international collaboration. It provides a macro-level strategic blueprint to guide future empirical, translational, and policy-oriented research at the intersection of artificial intelligence and nursing management. In addition, this study offers new insights through its broader temporal coverage (1990–2025), dual-database integration (Web of Science and Scopus), and the use of multiple bibliometric tools (VOSviewer, CiteSpace, Bibliometrix). These features enable a more comprehensive and fine-grained understanding of structural patterns, emerging themes, and collaboration dynamics in the field.

Bibliometric analysis applies mathematical and statistical methods to evaluate published literature on a specific topic, offering insights into publication trends, co-authorship, keyword frequency, and citation impact (17). It helps uncover influential studies, emerging themes, and collaboration networks—clarifying the development of a research field (18). In the context of artificial intelligence in nursing management, where research hotspots and developmental directions remain unclear, bibliometric analysis provides an effective means to map the field’s structure and highlight evolving trends. It allows researchers to trace the progression of knowledge, identify active contributors, and detect potential gaps. This study therefore conducts a bibliometric analysis of AI applications in nursing management, examining literature distribution across countries, institutions, authors, and journals, while analyzing keyword co-occurrence to detect thematic clusters. The goal is to guide future research directions and lay a foundation for academic progress in this interdisciplinary domain.

## 2 Materials and methods

### 2.1 Data sources and search strategy

**FIGURE 1:**
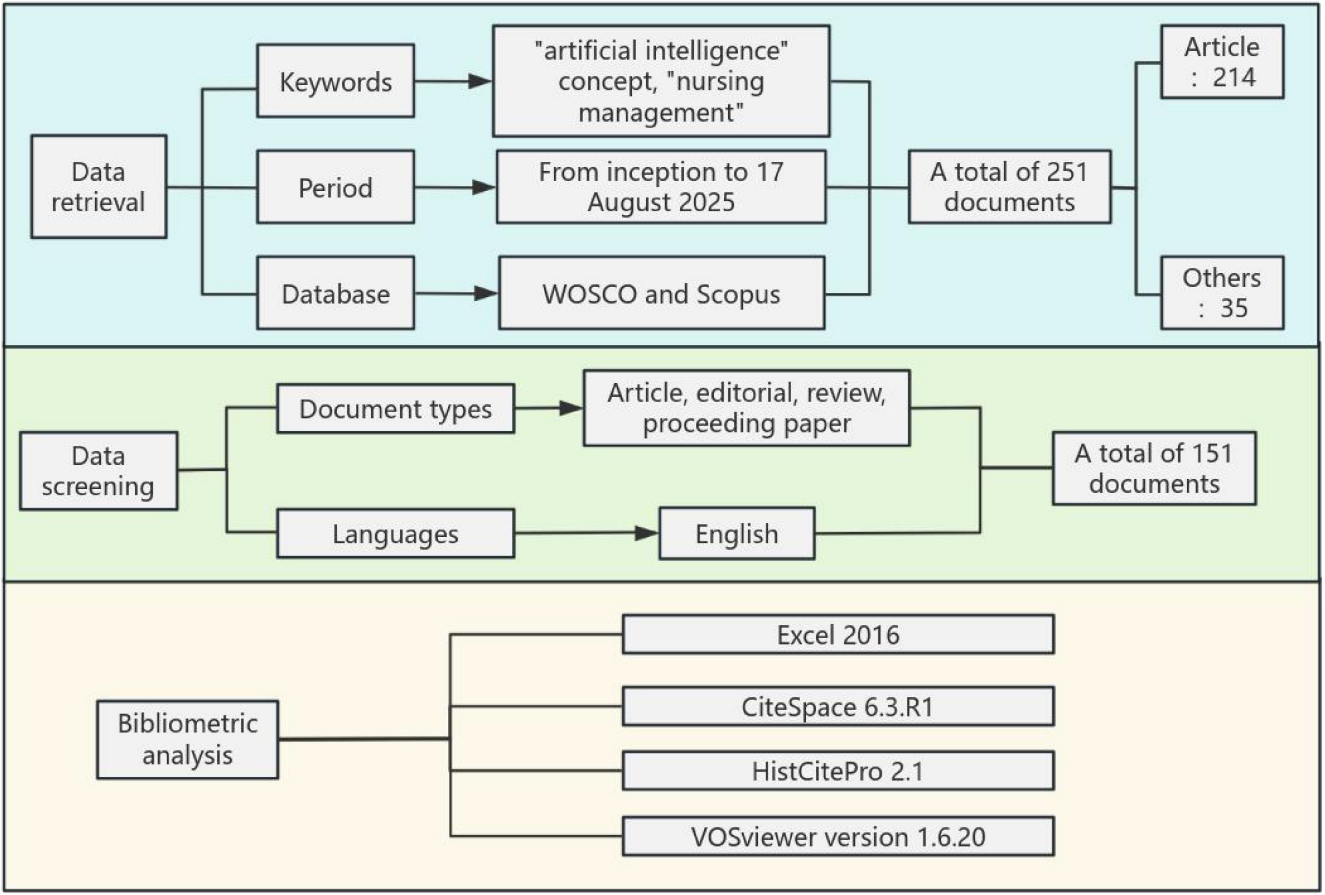
Flow chart of literature screening.

The Web of Science Core Collection (WOSCO) is one of the world’s most authoritative and widely used scientific literature databases, provided by Clarivate Analytics. The Scopus database stands as one of the world’s largest peer-reviewed abstract and citation databases, covering a broad spectrum of disciplines including natural sciences, technology, medicine, social sciences, and humanities. It ranks alongside WOSCO as an internationally authoritative academic literature database, primarily developed and operated by Elsevier in the Netherlands. Therefore, this study selected both databases as search sources, ultimately determining the literature retrieval formula in the WOSCO database as: TS = (“artificial intelligence” OR “AI” OR “machine intelligence” OR “machine learning” OR “deep learning” OR “data learning” OR “data mining” OR “big data” OR “intelligent systems” OR “intelligent learning” OR “feature* learning” OR “supervised learning” OR “supervised machine learning” OR “robot technology” OR “assistant robot” OR “robot*” OR “robot-assisted” OR “computational intelligence” OR “computer reasoning” OR “computer vision system” OR “sentiment analysis” OR “decision tree*” OR “mHealth” OR “e-health” OR “mobile health” OR “telehealth” OR “telemedicine” OR “digital health tools” OR “health information technology” OR “digital monitoring”) AND TS = (“nursing administration” OR “nurse* administration” OR “nursing management” OR “nurse* management” OR “nursing care management” OR “nursing management”). The literature search query in the Scopus database is: TITLE-ABS (“artificial intelligence” OR “AI” OR “machine intelligence” OR “machine learning” OR “deep learning” OR “data learning” OR “data mining” OR “big data” OR “intelligent systems” OR “intelligent learning” OR “feature* learning” OR “supervised learning” OR “supervised machine learning” OR “robot technology” OR “assistant robot” OR “robot*” OR “robot-assisted” OR “computational intelligence” OR “computer reasoning” OR “computer vision system” OR “sentiment analysis” OR “decision tree*” OR “mHealth” OR “e-health” OR “mobile health” OR “telehealth” OR “telemedicine” OR “digital health tools” OR “health information technology” OR “digital monitoring”) AND TITLE-ABS (“nursing administration” OR “nurse* administration” OR “nursing management” OR “nurse* management” OR “nursing care management” OR “nursing management”). The search period spanned from the database inception to August 17, 2025, yielding a total of 260 articles. To ensure data accuracy, two researchers independently reviewed the literature and applied the following inclusion and exclusion criteria. Inclusion criteria: ➀ Research content involved AI and nursing management. ➁ Language was English. Exclusion criteria: ➀ Literature types included conference abstracts, letters, or theses. ➁ Duplicate publications. ➂ Errata documents. Cross-checking was performed afterward, with a third researcher mediating disagreements. Ultimately, 151 English-language documents were included. The literature screening flowchart is shown in Figure 1.

### 2.2 Data standardization

The export of the qualifying publications “Full Record and Cited References” is performed in either “Plain Text File” format, with the filename “download*.txt.” The “Plain Text File” was loaded into CiteSpace, Bibliometrix, and VOSviewer for graphing. We first filtered the results according to our predefined search criteria in the WOSCO database and the Scopus database. To remove duplicate records between Web of Science and Scopus, we first merged the two datasets and used the built-in duplicate removal function in CiteSpace, which identifies overlapping entries based on a combination of metadata fields such as title, author, journal, and publication year. Records with identical or highly similar metadata were flagged as duplicates. After the automated process, two researchers manually reviewed the flagged records to confirm duplicates and resolve discrepancies. Only one version of each duplicated article was retained in the final dataset for analysis. To ensure consistency and accuracy in the subsequent analysis, synonyms were unified by creating a citespace. Alias file in CiteSpace and a thesaurus file in VOSviewer. In addition, irrelevant or meaningless entries such as “123” and “a” were excluded through a custom exclusion file.

### 2.3 Data analysis and visualization

This study employed bibliometric analysis to examine publications on the use of AI technology for nursing management. The analysis tools for this task were CiteSpace 6.3.R1, Bibliometrix, VOSviewer and Excel 2016, each serving specific analytical purposes.

CiteSpace is a Java-based scientific bibliometric and visualization analysis software developed by American scholar Chaomei Chen (19). It is widely used to identify knowledge frameworks, developmental trends, and emerging research hotspots within academic studies. In this study, CiteSpace was employed to integrate literature data from multiple databases. Through data integration and deduplication, information quality was enhanced.

Bibliometrix is an open-source bibliometric analysis tool developed by Aria and Cuccurullo et al. based on the R software (20). It can be used for citation analysis, co-occurrence analysis, collaboration network formation, and topic evolution identification. Biblioshiny, its graphical interface module, enables interactive operations and result visualization, making it widely applied in scientific knowledge graph construction and research performance evaluation studies.

VOSviewer is a bibliometric software tool developed in 2010 by Nees Jan van Eck and Ludo Waltman at Leiden University (21). While it is primarily designed for the analysis and visualization of bibliometric networks derived from academic literature, its utility extends to any type of network data. The software enables the construction of co-occurrence networks based on specific terms, where the spatial distance and color of nodes represent the strength of association and temporal distribution, respectively. This facilitates the identification of structural patterns and emerging trends within a given research domain. In this study, VOSviewer was used to perform network visualization mapping based on countries/regions, institutions, authors, journals, references, keywords and calculate the centrality of countries/regions. Visual network diagrams were generated to present the overall landscape, research hotspots, and development trends of AI applications in nursing management studies in a clear and intuitive manner, providing valuable reference for future research. Line thickness between nodes indicates collaboration intensity—thicker lines signify stronger collaboration and higher corresponding Total Link Strength (TLS). VOSviewer was also used to analyze TLS.

The thresholds of VOSviewer were set as follows—countries/regions collaboration network: countries with a minimum of 2 paper; institutions cooperation network: institutions with a minimum of 1 paper; author collaboration network: authors with a minimum of 1 paper; co-cited authors network: authors with a minimum of 1 citation; co-cited journals network: journals with a minimum of 10 citations; references citation network: references with a minimum of 1 citation; co-cited references network: references with a minimum of 2 citations; keywords co-occurrence network: keywords with a minimum of 3 occurrences. The clustering algorithm is based on modularity optimization and VOS (Visualization of Similarities) mapping technique developed by Van Eck and Waltman (21). Default resolution parameters were used.

The H-index was adopted as a quantitative metric to assess the academic influence of scholars, institutions, and journals (22). The Impact Factor (IF) is a commonly used metric for measuring the influence of academic journals, published annually by Clarivate. It represents the average number of times articles published in a journal over the past 2 years are cited in the current year (23). Journal Citation Reports (JCR) is a database published by Clarivate Analytics specifically designed to evaluate the impact and citation metrics of academic journals worldwide. It serves as a vital tool for measuring the scholarly quality and influence of journals (24). IF, JCR and H-index for 2024 were also obtained from the Web of Science. What’s more, Excel 2021 was used to plot bar charts of publication volumes and perform exponential fitting.

## 3 Results

### 3.1 Overview of main information and annual publications trend

**FIGURE 2:**
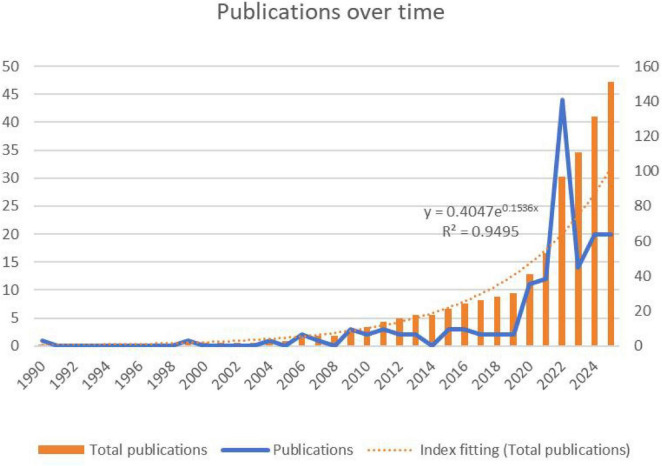
Trends in annual publications.

A total of 151 articles were included in the literature review, comprising 214 original research articles (85.94%) and 20 review articles (8.03%). For details, please refer to Supplementary material. Academic interest in AI applications for nursing management first emerged in 1990 with only one published paper. The trend can roughly be divided into three stages. From 1990 to 2009, research remained in its infancy with negligible output and limited scholarly attention. Between 2010 and 2016, the field entered a period of gradual growth, with small but steady year-on-year increases. Since 2017, research has accelerated rapidly, with annual publication volume rising sharply and peaking in 2021 at 45 publications. Although output fell to 15 in 2023, it rebounded to 20 in 2024–2025, and overall productivity remains high. Notably, publications in the past 4 years (2021–2024) account for about two-thirds (∼67%) of the total, reflecting strong recent interest. Total publications exhibit a clear exponential growth trend. The fitted exponential curve (*y* = 0.4047e^0.1536x^ shows *R*^2^ = 0.9495, indicating a strong correlation between publication year and output. These findings suggest that this field has evolved from an emerging topic into a rapidly expanding area of sustained academic interest, as shown in Figure 2.

### 3.2 Distribution of countries/regions and institutions

A total of 43 countries/regions contributed to the research in this field. The country with the highest number of publications was China (54 publications), followed by the USA (29 publications), Canada (8 publications), Finland (7 publications), Italy (6 publications), South Korea (6 publications), Taiwan (6 publications), Spain (5 publications), Brazil (4 publications) and the Australia (3 publications). The top 10 country/region accounted for 84.8% of the total publications in this field. A detailed breakdown of the publication numbers from these top 10 countries/regions is shown in Table 1.

**TABLE 1:** The top 10 countries/regions of publication volume.

Rank	Countries/regions	Publications	Percentage	TLS	First appearance time
1	China	54	35.8	12	2016
2	USA	29	19.2	15	1990
3	Canada	8	5.3	5	2022
4	Finland	7	4.6	4	2004
5	Italy	6	4.0	1	2022
6	South Korea	6	4.0	1	2006
7	Taiwan	6	4.0	1	2015
8	Spain	5	3.3	2	2024
9	Brazil	4	2.6	1	2020
10	Australia	3	2.0	1	2016

TLS, Total Link Strength.

**FIGURE 3:**
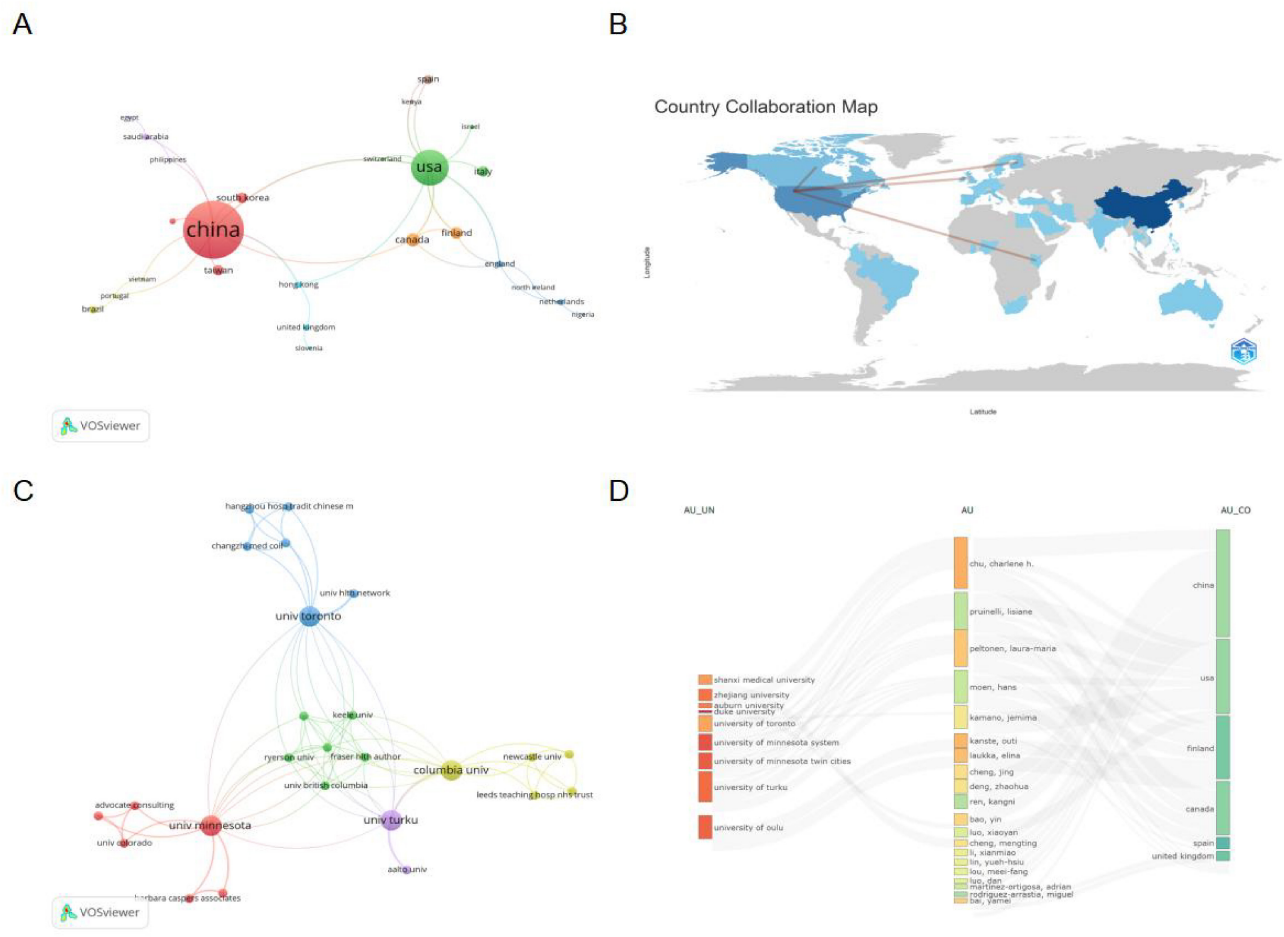
(A) Network visualization of collaboration among countries/regions. (B) Country collaboration map. (C) Network visualization of collaboration among institutions. (D) Three-field plot visualization the relationships between institutions, authors, and countries.

Figure 3A displays the collaborative network among countries/regions based on VOSviewer. Node size represents the number of publications, while line thickness between nodes reflects collaboration intensity. The United States and China serve as central nodes in this network, engaging in significant collaboration both between themselves and with countries such as the United Kingdom, Finland, and South Korea. Figure 3B presents a country collaboration map, color-coded according to each nation’s collaboration level. The figure highlights China’s robust partnerships with North American and European countries, with the United States and China forming critical links that drive the majority of research output in this field.

In terms of institutional collaboration, a total of 344 institutions participated in research on AI applications in nursing management. Table 2 lists the top 10 institutions by number of published papers. Among these, half of the institutions are from the United States, while two Chinese institutions made the top 10: Capital Medical University and Anhui University of Science and Technology. The institutional collaboration network analyzed using VOSviewer is shown in Figure 3C. This map illustrates how institutions across different regions collaborate on research. Institutions from the United States, including University of Minnesota System and the Columbia University, emerge as prominent nodes in the network, reflecting their central roles in the field. The network also highlights significant collaborations with European and American institutions, particularly those in countries like Finland and Canada.

**TABLE 2:** The top 10 institution of publication volume.

Rank	Institution	Country	Publications	TLS
1	Capital Medical University	China	3	2
2	Columbia University	USA	3	3
3	University of Minnesota System	USA	3	3
4	University of Toronto	Canada	3	3
5	University of Turku	Finland	3	3
6	Anhui University of Science and Technology	China	2	1
7	Auburn University	USA	2	2
8	Augusta University	USA	2	2
9	Duke University	USA	2	2
10	Icahn School of Medicine at Mount Sinai	USA	2	2

TLS, Total Link Strength.

Figure 3D presents a three-field plot illustrating relationships among institutions, authors, and countries. This plot demonstrates how specific institutions collaborate with authors from diverse regions, highlighting the international character of the research. Key institutions such as University of Minnesota System, University of Turku, and University of Toronto are displayed alongside their respective collaborators, revealing strong connections with researchers from USA, China, and Finland.

### 3.3 Authors and co-cited authors analysis

**FIGURE 4:**
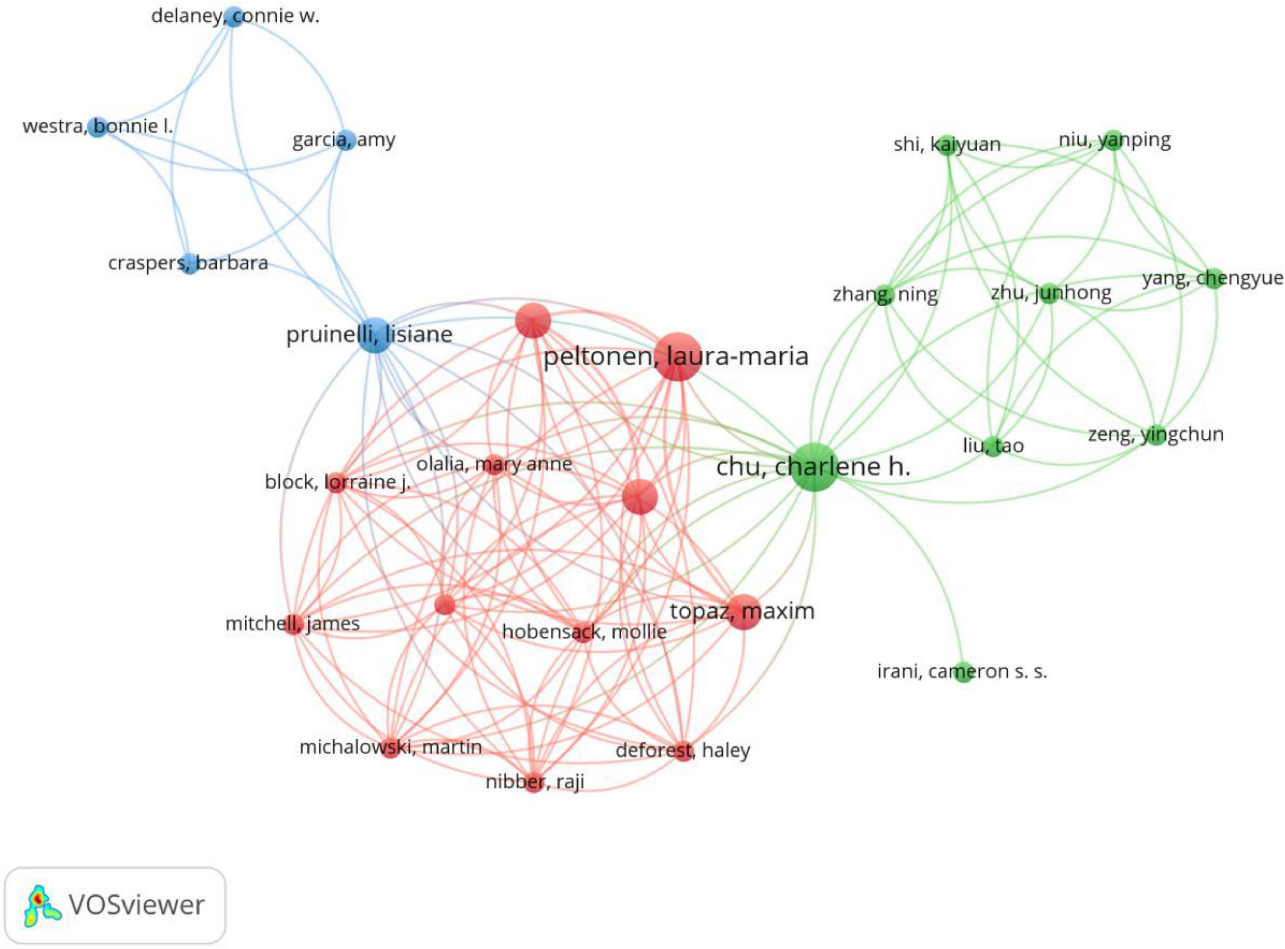
Network visualization of collaboration among authors.

A total of 698 authors have contributed to publications in this research field. The top 10 most productive authors are listed in Table 3, among whom Charlene H. Chu and Laura-Maria Peltonen are the leading contributors. The data in the table reveals that despite publishing only 3 articles, Charlene H. Chu has garnered a significant 179 citations, indicating her substantial academic influence in this field. In contrast, authors with similar publication counts, such as Yin Bao and Jing Cheng, who each published 2 articles, received no citations each, suggesting their relatively lower academic impact. The author collaboration network is shown in Figure 4, which reveals three major clusters of cooperation. Overall, the network exhibits a multi-centered structure dominated by three major clusters: red, blue, and green. The red cluster forms the core of the network, represented by Laura-Maria Peltonen, with dense connections among its members—such as Maxim Topaz; Lorraine J. Block; and James Mitchell—indicating strong collaborative relationships. The blue cluster, centered around Lisiane Pruinelli, includes scholars such as Connie W. Delaney, Bonnie L. Westra, and Amy Garcia. While their collaborative relationships are close, they remain relatively independent. The green cluster comprises scholars like Junhong Zhu, Chengyue Yang, and Ning Zhang, revealing a distinctly regional pattern of researcher collaboration. Notably, Charlene H Chu. locates between the red and green groups, featuring a larger node size and numerous connections. This indicates her high intermediary centrality within the network, serving as a vital bridge connecting diverse academic communities and fostering international collaboration.

**TABLE 3:** The top 10 authors of publication volume.

Rank	Author	Publications	Citations	TLS	H-index
1	Chu, Charlene H.	3	179	21	20
2	Kanste, Outi	3	41	6	16
3	Laukka, Elina	3	41	6	11
4	Peltonen, Laura-Maria	3	143	16	13
5	Bai, Yamei	2	1	19	5
6	Bao, Yin	2	0	7	3
7	Cheng, Jing	2	0	6	1
8	Cheng, Mengting	2	19	4	5
9	Deng, Zhaohua	2	0	6	28
10	Kamano, Jemima	2	54	24	20

TLS, Total Link Strength.

**FIGURE 5:**
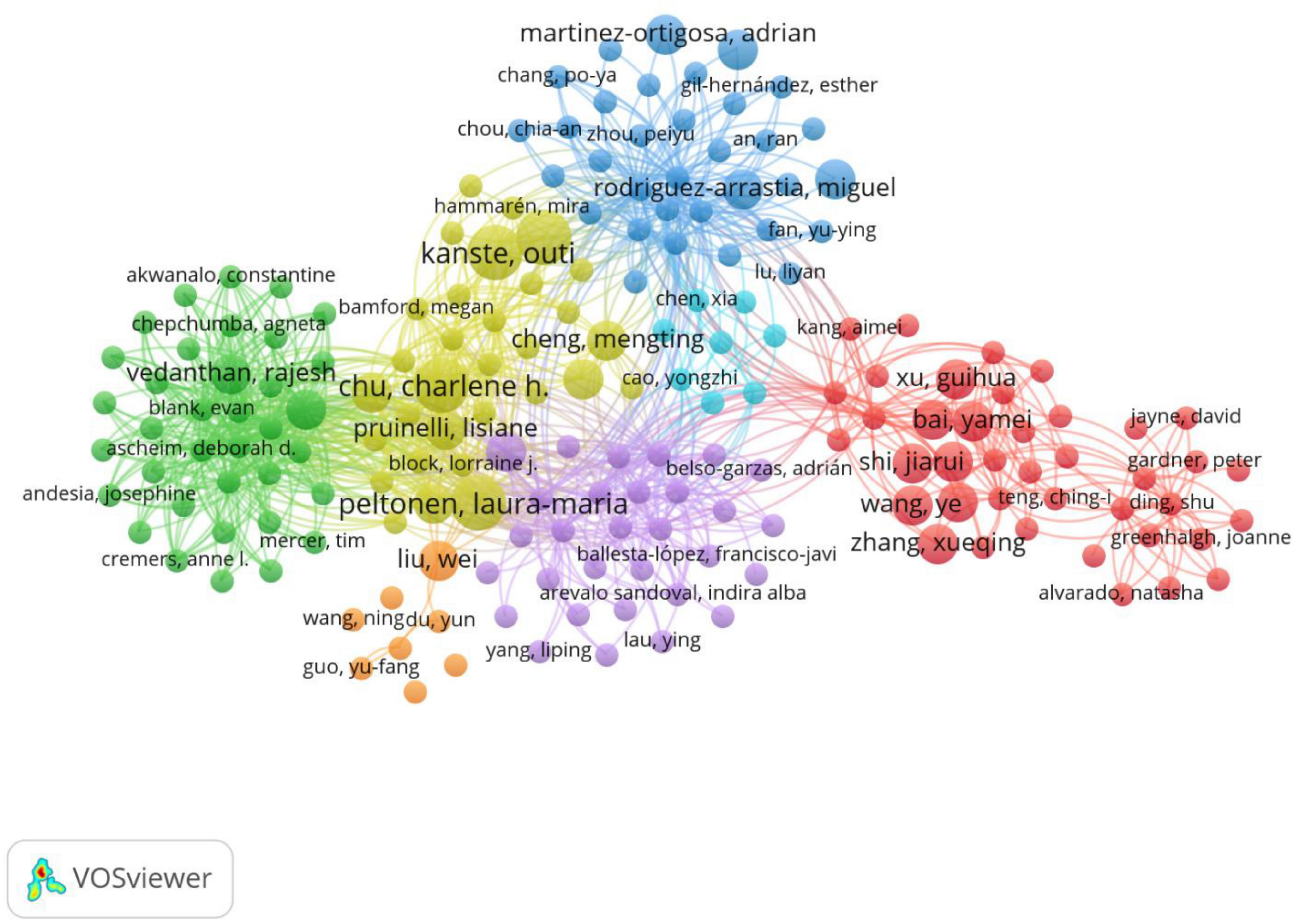
Network visualization of co-cited authors.

The top 10 most co-cited authors are listed in Table 4. The analysis of co-cited authors in this study highlights key scholars who have made significant contributions to the research field. Table 4 presents the top 14 co-cited authors, with Charlene H. Chu leading with 179 citations and a TLS of 30, indicating his central role in academic collaboration within the field. Other highly co-cited authors, such as Lisiane Pruinelli (149 citations, TLS = 30) and Laura-maria Peltonen (143 citations, TLS = 49), also demonstrate strong academic influence, with high citation counts and substantial collaborative ties. The co-cited author network, as shown in Figure 5, visually represents the interconnectedness of these key authors. The network is characterized by multiple clusters, where authors with strong co-citation relationships are grouped together. These clusters reflect the diversity of research directions and areas of influence within the field of AI applications in nursing management. The network also illustrates the prominent position of several authors, with Laura-maria Peltonen standing out with the highest TLS of 49, suggesting his active involvement in numerous academic collaborations.

**TABLE 4:** The top 14 co-cited authors of publication volume.

Rank	Author	Citations	TLS
1	Chu, Charlene H.	179	30
2	Pruinelli, Lisiane	149	30
3	Peltonen, Laura-Maria	143	49
4	Moen, Hans	139	42
5	Von Gerich, Hanna	139	42
6	Topaz, Maxim	138	37
7	Block, Lorraine J.	134	30
8	Deforest, Haley	134	30
9	Hobensack, Mollie	134	30
10	Michalowski, Martin	134	30
11	Mitchell, James	134	30
12	Nibber, Raji	134	30
13	Olalia, Mary Anne	134	30
14	Ronquillo, Charlene E.	134	30

TLS, Total Link Strength.

### 3.4 Journal and cited journal analysis

The journal impact factor (IF) and the Journal Citation Reports (JCR) quartile rankings serve as indicators of a journal’s academic influence. Journals within the top 25% of impact factors (including the 25th percentile) are classified as Quartile 1 (Q1), while those ranked between the top 25% and 50% (including the 50th percentile) fall into Quartile 2 (Q2). Journal analysis facilitates the recognition of core journals that play a central role in a given academic discipline (25). A total of 80 journals contributed to research in this field and an online study was conducted on the 2024 Impact Factor (IF) and Journal Citation Reports (JCR) partitions. The top 14 journals with the highest number of publications on AI applications in nursing management are shown in Table 5. Journal of Nursing Management leads in publication volume with 53 articles, holds an impact factor of 4, and is ranked in Q1 of the JCR, indicating its significant academic influence in the field. Other journals such as International Journal of Medical Informatics (IF = 4.1) and JMIR Nursing (IF = 4.0) also hold prominent positions in nursing and medical informatics. Most of the top 10 journals are in Q1 of the JCR, reflecting the high level of attention AI research is receiving in nursing management. Meanwhile, Q2 journals like CIN-Computers Informatics Nursing and Journal of Healthcare Engineering, despite their lower impact factors, demonstrate interdisciplinary interest in this topic.

**TABLE 5:** The top 14 most prolific journals of publication volume.

NO	Journal	Publications	IF (2024)	JCR (2024)
1	Journal Of Nursing Management	53	4	Q1
2	Journal Of Healthcare Engineering	6	3.822 (2021)	Q2 (2021)
3	Cin-Computers Informatics Nursing	3	1.9	Q2
4	BMC Health Services Research	2	3	Q1
5	BMC Nursing	2	3.9	Q1
6	Computational Intelligence and Neuroscience	2	3.12 (2021)	Q2 (2021)
7	Geriatric Nursing	2	2.4	Q2
8	International Journal of Medical Informatics	2	4.1	Q1
9	JMIR Nursing	2	4	Q1
10	Journal of Clinical Nursing	2	3.5	Q1
11	Journal of Nursing Scholarship	2	2.9	Q1
12	Journal of Robotic Surgery	2	3	Q1
13	Nursing Open	2	2.3	Q1
14	Nursing Reports	2	2	Q1

JCR, Journal Citation Reports; IF, Impact Factor.

**FIGURE 6:**
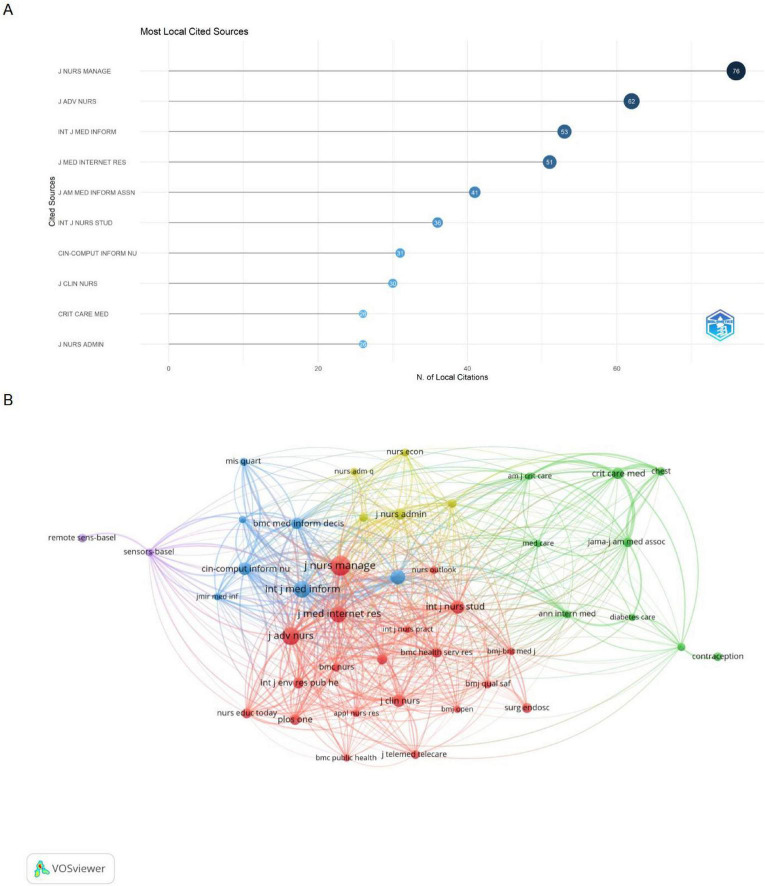
(A) Bar chart of top 10 most co-cited journals. (B) Network visualization of co-cited journals.

In this study, a total of 3603 co-cited journals were identified. Table 6 lists the top 10 journals by co-citation frequency, with Journal of Nursing Management leading at 76 citations, an Impact Factor of 4, and JCR Q1 ranking. It is followed by Journal of Advanced Nursing (62 citations, IF = 3.4, Q1) and International Journal of Medical Informatics (79 citations, IF = 6, Q1). Other top-10 journals include the International Journal of Medical Informatics (64 citations, IF = 4.1, Q1), Journal of Clinical Nursing (56 citations, IF = 3.5, Q1), and International Journal of Nursing Studies (54 citations, IF = 7.1, Q1), indicating that AI research in nursing management is concentrated in high-impact nursing and medical informatics journals. Bar chart of top 10 most co-cited journals and network visualization of co-cited journals were presented in Figure 6.

**TABLE 6:** The top 10 most co-cited journals.

NO	Journal	Citations	IF (2024)	JCR (2024)
1	Journal of Nursing Management	76	4	Q1
2	Journal of Advanced Nursing	62	3.4	Q1
3	International Journal of Medical Informatics	53	4.1	Q1
4	Journal of Medical Internet Research	51	6	Q1
5	Journal of the American Medical Informatics Association	41	4.6	Q1
6	International Journal of Nursing Studies	36	7.1	Q1
7	Cin-Computers Informatics Nursing	31	1.9	Q2
8	Journal of Clinical Nursing	30	3.5	Q1
9	Clinical and Translational Medicine	26	4.614 (2021)	Q1(2021)
10	Journal Of Nursing Administration	26	2.9	Q1

JCR, Journal Citation Reports; IF, Impact Factor.

### 3.5 Reference citation analysis

**FIGURE 7:**
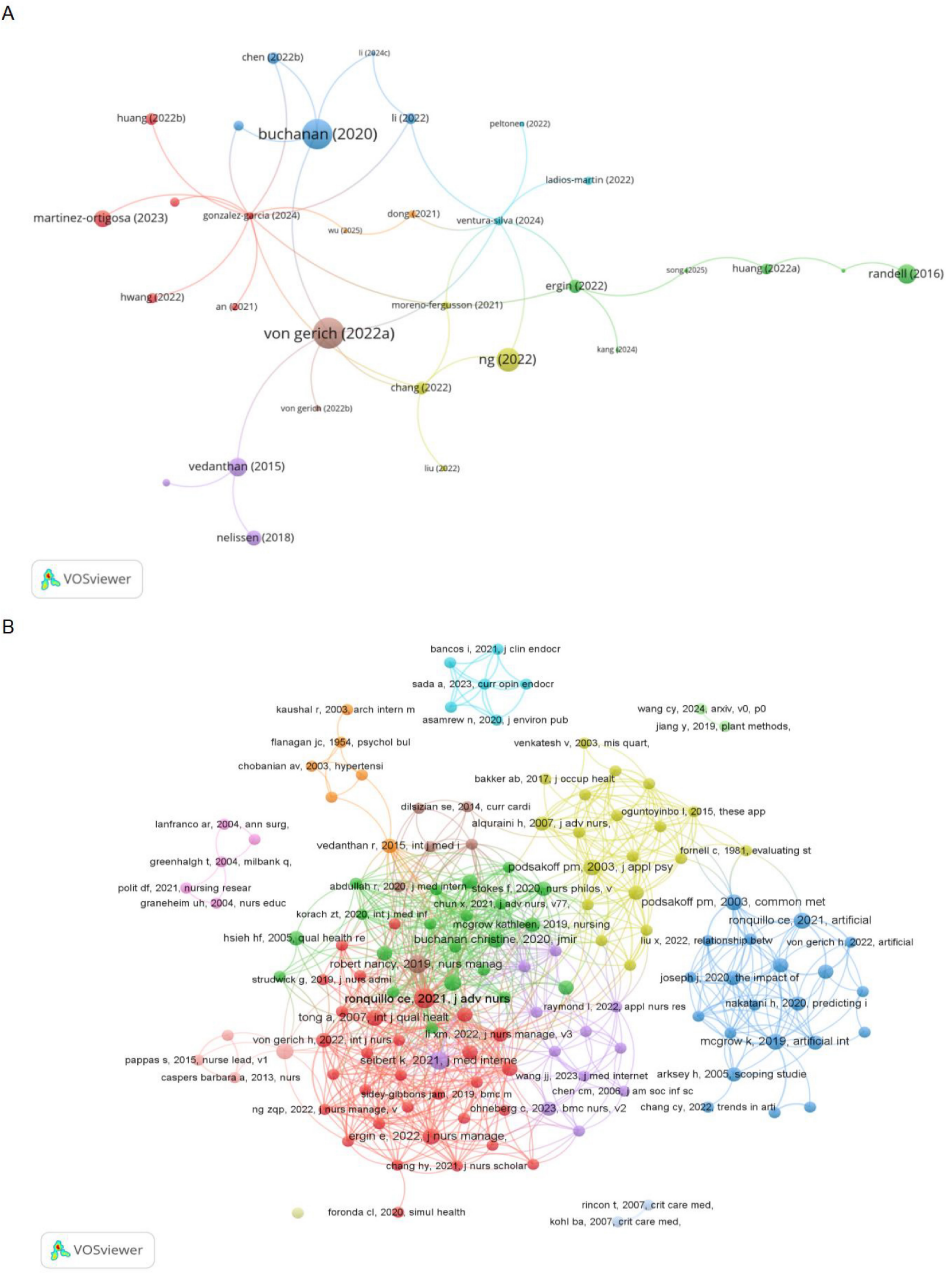
(A) Network visualization of reference citation. (B) Network visualization of co-cited references.

By analyzing the most frequently cited references in the field, a center and focus for research can be identified, thereby helping beginners in the field quickly grasp the research process and direction (26). The number of times the 136 documents were cited was determined by VOSviewer, and the top 10 most frequently cited documents are displayed in Table 7, and the network visualization of reference citation was displayed in Figure 7A. “Artificial Intelligence-based technologies in nursing: A scoping literature review of the evidence” (15), published in International Journal of Nursing Studies, received the most citations of 134.

**TABLE 7:** The top 10 most frequently cited documents.

NO	Journal	Citations	IF (2024)	JCR (2024)
1	Artificial Intelligence-based technologies in nursing: a scoping literature review of the evidence	International Journal of Nursing Studies	2022	134
2	Predicted influences of artificial intelligence on the domains of nursing: scoping review	JMIR Nursing	2020	126
3	Precision health: a nursing perspective	International journal of Nursing Sciences	2020	85
4	The role of artificial intelligence in enhancing clinical nursing care: a scoping review	Journal of Nursing Management	2021	74
5	Embedding robotic surgery into routine practice and impacts on communication and decision making: a review of the experience of surgical teams	Cognition, Technology& Work	2016	50
6	Usability and feasibility of a tablet-based Decision-Support and Integrated Record-keeping (DESIRE) tool in the nurse management of hypertension in rural western Kenya	International Journal of Medical Informatics	2015	46
7	A comparison between Pixel-based deep learning and Object-based image analysis (OBIA) for individual detection of cabbage plants based on UAV Visible-light images	Computers and Electronics in Agriculture	2023	44
8	Ethical issues of smart home-based elderly care: a scoping review	Journal of Nursing Management	2021	41
9	Applications of artificial intelligence in nursing care: a systematic review	Journal of Nursing Management	2023	41
10	Reducing cardiovascular disease risk in medically underserved urban and rural communities	American Heart Journal	2011	36

The results show a total of 5410 co-cited references, Table 8 listed the top 10 most co-cited references. “Artificial intelligence in nursing: Priorities and opportunities from an international invitational think-tank of the Nursing and Artificial Intelligence Leadership Collaborative” (27) was the most co-cited reference. The visualized network of co-cited references was created via VOSviewer and displayed in Figure 7B.

**TABLE 8:** The top 10 most co-cited reference.

Rank	Title	Journal	Year	Citations
1	How artificial intelligence is changing nursing	Nursing Management	2019	20
2	Artificial intelligence in nursing: Priorities and opportunities from an international invitational think-tank of the Nursing and Artificial Intelligence Leadership Collaborative	Journal of Advanced Nursing	2021	18
3	Common method biases in behavioral research: a critical review of the literature and recommended remedies	Journal of Applied Psychology	2003	13
4	Application Scenarios for Artificial Intelligence in Nursing Care: Rapid Review	Journal of Medical Internet Research	2021	12
5	Scoping studies: toward a methodological framework	International Journal of Social Research Methodology	2005	11
6	Artificial intelligence: Essentials for nursing.	Nursing	2019	11
7	Consolidated criteria for reporting qualitative research (COREQ): a 32-item checklist for interviews and focus groups	International Journal For Quality in Health Care	2007	11
8	Predicted Influences of Artificial Intelligence on the Domains of Nursing: Scoping Review	Jmir Nursing	2020	10
9	Artificial Intelligence -based technologies in nursing: A scoping literature review of the evidence.	International journal of nursing studies	2022	10
10	PRISMA Extension for Scoping Reviews (PRISMA-ScR): Checklist and Explanation	Annals of Internal Medicine	2018	8
11	Identification of important factors in an inpatient fall risk prediction model to improve the quality of care using ehr and electronic administrative data: a machine-learning approach	International Journal of Medical Informatics	2020	8
12	the role of artificial intelligence in enhancing clinical nursing care: a scoping review	Journal of Nursing Management	2022	8

### 3.6 Keywords analysis

An overview of central components through keywords analysis aims to identify key aspects of AI in nursing management research (28). Table 9 shows the top 20 keywords by frequency. As shown in the table, keywords such as “nursing,” “nursing management,” “health care,” “AI,” “machine learning,” and “deep learning” appear frequently and occupy central positions within the network, indicating their significant role in research integrating nursing with intelligent technologies.

**TABLE 9:** The top 20 keywords by frequency.

Rank	Keywords	Frequency	TLS	Rank	Keywords	Frequency	TLS
1	AI	31	131	11	Nursing care	7	42
2	Nursing management	21	166	12	Procedures	7	57
3	Telemedicine	21	112	13	Cross-sectional study	6	58
4	Health care	15	112	14	Decision making	6	28
5	Machine learning	10	39	15	Diagnosis	6	34
6	Big data	9	40	16	Electronic health record	6	28
7	Questionnaire	9	77	17	Impact	6	23
8	Surveys and questionnaires	8	75	18	Information management	6	34
9	Technology	8	28	19	Medical information	6	60
10	Data mining technology	7	42	20	Middle aged	6	59

TLS, Total Link Strength.

**FIGURE 8:**
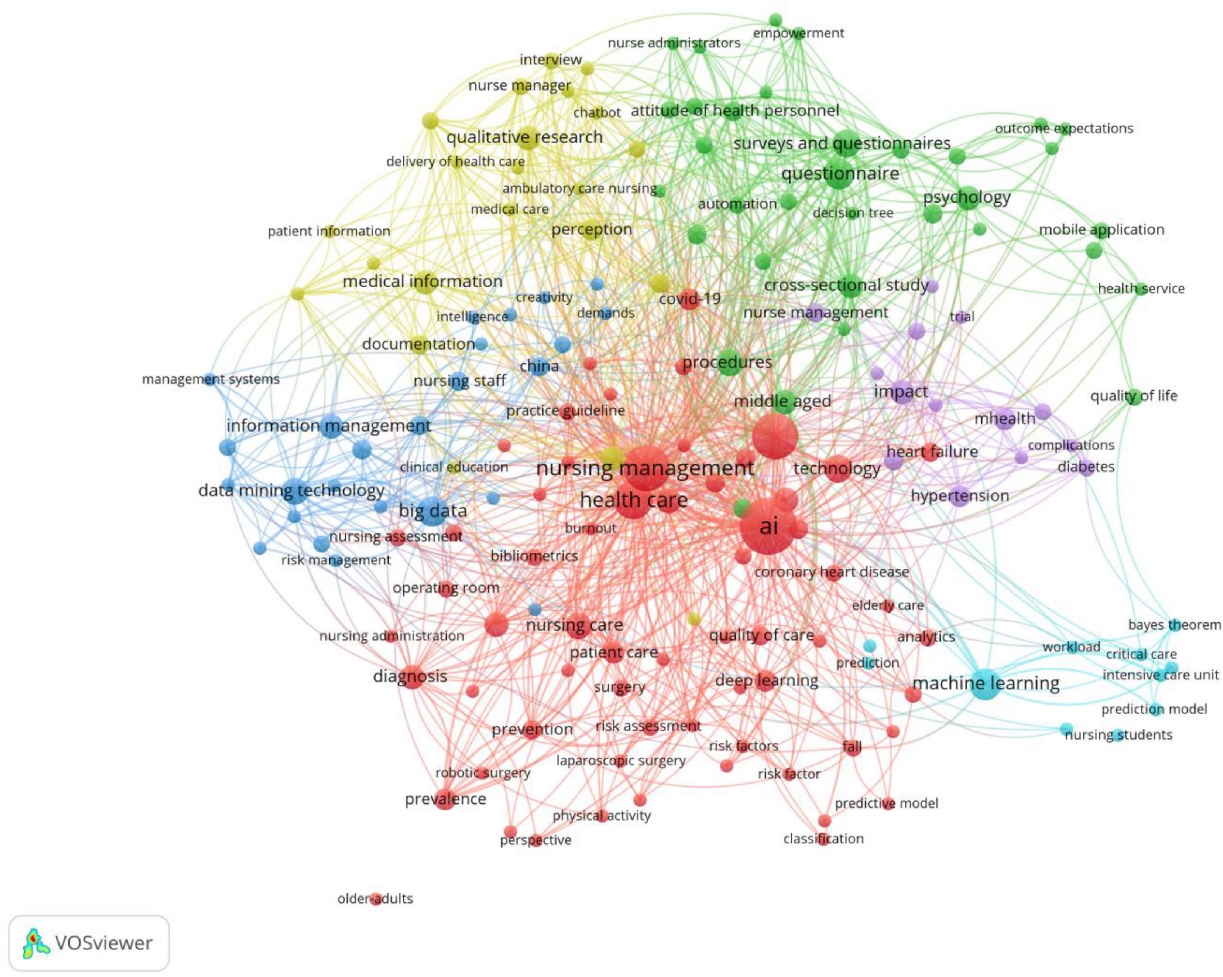
Network visualization of keywords.

After removing non-informative keywords, we constructed a network comprising 170 keywords that appeared at least 2 times in VOSviewer, resulting in five distinct clusters (Figure 8). The nodes in the figure represent keywords, with node size reflecting their frequency of occurrence. Connecting lines indicate co-occurrence relationships between keywords, while different colors denote distinct clustering modules. Based on the clustering results, keywords can be generally classified into the following five research themes: (1) Nursing Management and Clinical Application (Red Cluster): Representative keywords include “nursing management,” “health care,” “AI,” “deep learning,” “machine learning,” “risk assessment,” and “quality of care.” This cluster represents the core area of research, focusing on integrating artificial intelligence into nursing management and clinical decision-making. Studies in this domain emphasize AI-based prediction, risk assessment, and optimization of nursing workflows to enhance patient safety and quality of care. (2) Psychological and Behavioral Research (Green Cluster): Representative keywords include “survey,” “questionnaire,” “psychology,” “attitude of health personnel,” and “quality of life.” This cluster highlights the social and behavioral aspects of AI adoption, including nurses’ perceptions, attitudes, and psychological responses to digital transformation. It reflects growing interest in the human and ethical dimensions of implementing AI in healthcare systems. (3) Information Management and Big Data Technology (Blue Cluster): Representative keywords include “information management,” “data mining technology,” “big data,” “risk management,” and “documentation.” This cluster focuses on the technological infrastructure supporting AI applications in nursing, particularly the use of data analytics, information systems, and digital records to inform evidence-based nursing management. (4) Managerial and Qualitative Research (Yellow Cluster): Representative keywords include “qualitative research,” “nurse manager,” “interview,” “empowerment,” and “delivery of health care.” This cluster reflects organizational and leadership perspectives, exploring how nurse managers and healthcare administrators perceive and implement AI technologies. Studies in this area often use qualitative approaches to examine managerial readiness, staff training, and innovation capacity in nursing organizations. (5) Clinical Disease Prediction and AI Modeling (Purple/Cyan Cluster): Representative keywords include “machine learning,” “prediction model,” “heart failure,” “hypertension,” “diabetes,” and “critical care.” Overall, these five clusters reveal that research on AI in nursing management is characterized by strong interdisciplinarity, combining technological innovation, data-driven methods, human factors, and managerial strategies to improve the efficiency and quality of healthcare delivery.

## 4 Discussion

This bibliometric analysis comprehensively presents the research landscape of AI in nursing management from 1990 to the present (data up to August 2025), revealing a field that has evolved from an emerging discipline into a rapidly expanding research frontier. The exponential growth in the number of publications, particularly after 2017, reflects the academic community’s rising interest in this field. This growth is driven by both technological readiness and pressing clinical needs. Notably, the average citation metrics and H-index of leading authors indicate a shift in research focus from exploratory studies to evidence-based, practice-oriented research with significant academic impact. The most critical development occurred around 2017, when machine learning—particularly deep learning—achieved substantial breakthroughs in image recognition, natural language processing, and other areas (14). Medical AI products have gradually transitioned from the “experimental phase” to “actual deployment,” spurring a surge in empirical research across domains such as nursing decision support, health monitoring, and scheduling management (29). These advancements enable intelligent systems to be applied in real-world care settings—for tasks like automated documentation, risk prediction, shift optimization, and decision support—transforming AI from a conceptual innovation into an essential practical component within nursing management workflows (30, 31).

Among the 43 contributing countries/regions, the top 10 countries/regions accounted for 84.8% of all publications, indicating that this field is predominantly driven by a handful of highly productive countries/regions. China leads with 54 publications (35.8%), reflecting its ongoing investments in healthcare digitization and extensive healthcare infrastructure. Despite publishing fewer publications (31), the USA holds a TLS of 15, indicating its pivotal role in international collaboration and academic influence. Countries/regions visualization collaboration network diagrams reveal that the USA and China lie a central position within the cooperative network at the national level. One possible reason for this situation is that the USA began research in this field as early as 1990 (32), accumulating extensive experience and playing a central role in global academic collaboration. China, however, only entered a period of rapid development starting in 2016 (33), becoming a major global contributor to research. Its research achievements have primarily focused on the Asian region, maintaining close cooperative relationships with countries such as South Korea and Taiwan. Not only have the two countries engaged in frequent collaboration with each other, but they have also established close ties with nations such as the UK, Finland, and South Korea. Especially after 2020, their research activities in the field of AI-driven nursing management have significantly increased. This collaboration demonstrates that global scientific cooperation is accelerating, particularly in the medical field where substantial funding and technical support are required. Through international cooperation, resources, data, and expertise can be shared among countries, accelerating technological innovation and application (34).

A bibliometric analysis of institutions reveals that USA’s institutions dominate research in this field, particularly Columbia University and University of Minnesota System. These institutions play a central role in global collaborative networks and demonstrate outstanding performance in both publication volume and TLS. This situation likely stems primarily from the USA’s longstanding global leadership in AI technology research and application. This advantage enables American institutions to secure critical academic resources, technical support, and international collaboration opportunities within the AI nursing management field (35). Moreover, the U.S. Government’s supportive policies have also played a crucial role in advancing the development of artificial intelligence technology (36). For example, the U.S. Government has vigorously supported the development of AI technology by establishing and implementing large-scale scientific research funding programs, such as the funding initiatives of DARPA and NSF, while encouraging interdisciplinary collaborative research. Such policy safeguards enable the rapid application of U.S. AI technology and research outcomes to practical problems, including healthcare and nursing management (37).

Among the top ten institutions by publication volume, two Chinese universities, Capital Medical University and Anhui University of Science and Technology, have achieved notable research output. However, their collaboration intensity and influence remain relatively low, indicating that China’s AI nursing management research still needs improvement. This may reflect systemic factors in China’s research ecosystem, such as government emphasis on rapid publication output under initiatives like Healthy China 2030 and the New Generation Artificial Intelligence Development Plan, which prioritize scaling up national digital health infrastructure (35). Moreover, academic performance metrics in China often emphasize quantity over international engagement or research impact, potentially limiting cross-border collaboration (38). Language barriers and regional clustering in research networks may also contribute to lower global visibility. Thus, while China leads in publication volume, the USA maintains leadership in international integration, research influence, and technological translation capacity. These contrasting strengths underscore the need for more balanced global collaboration and capacity building in the field.

Author analysis in this field indicates that Charlene H. Chu, Outi Kanste, Elina Laukka and Laura-Maria Peltonen are the leading contributors, each having published 3 articles. Charlene H. Chu ranks among the top three authors by publication volume, yet her citation count significantly exceeds others at 179, with a TLS of 21, indicating her pivotal role in academic collaboration. Additionally, authors such as Outi Kanste and Elina Laukka exhibit high publication frequencies, demonstrating the field’s extensive and in-depth scholarly activity. In contrast, authors like Yamei Bai and Yin Bao, despite having published 2 articles, received no citations, indicating their relatively lower academic impact. This discrepancy underscores the distinction between publication volume and academic influence, which is more accurately reflected in citations and TLS. Further analysis of the author collaboration network reveals that there are distinct research directions within the field of AI applications in nursing management. Western scholars such as Laura-Maria Peltonen (15), Maxim Topaz (39), and Lorraine J. Block (15) focus on optimizing AI in nursing management, with particular attention to enhancing service efficiency, decision support, and automating nursing processes. Conversely, Chinese scholars like Junhong Zhu (40), Chengyue Yang (40), and Ning Zhang (41) primarily concentrate on leveraging AI for improving nursing quality, particularly in intelligent health management and nursing staff training. The collaboration between Western and Chinese scholars highlights the global nature of research in this field, with scholars like Charlene H. Chu playing a vital role as intermediaries who foster cross-regional collaborations, thereby facilitating the sharing of AI technologies in global nursing management systems. Additional research centers on data analysis and optimization, such as studies by James Mitchell (42) and Lorraine J. Block (43) exploring how AI enables real-time monitoring of patient health data and the development of personalized care plans. Co-cited author network analysis reveals academic collaboration patterns and influence of AI in nursing management. Core scholars such as Eliasu Abdulai (44) and Constantine Akwanalo (45), despite limited publications, serve as hubs in the academic network due to their high citation counts and robust collaborative ties. This indicates that academic influence depends more on the breadth and depth of collaborative networks than on sheer publication volume. Additionally, network analysis uncovers trends in interdisciplinary and cross-regional collaboration. Western scholars focus on optimizing AI applications in nursing management, while researchers from China and other Asian nations make significant contributions to enhancing nursing quality and health management. Despite expanding global academic cooperation, geographical and disciplinary barriers persist, particularly with relatively low academic participation from Africa. Future research can advance global academic exchange and technology transfer through international collaboration platforms and interdisciplinary cooperation, thereby enhancing the effectiveness of AI applications in nursing management.

The present study explored the academic influence and collaborative networks of AI in nursing management through journal and co-cited journal analysis. Findings revealed that numerous core journals, such as the Journal of Nursing Management (46), BMC Health Services Research (45), and BMC Nursing (47) and so on, are ranked in JCR Q1 with high impact factors, indicating their significant academic influence in this field (46). Notably, the Journal of Nursing Management, which published the highest number of publications, further solidified its academic leadership. These high-impact journals not only provide crucial platforms for AI applications in nursing management but also foster global academic attention through elevated citation frequencies and impact factors. Furthermore, the findings underscore the critical role of interdisciplinary collaboration. With the rapid advancement of AI technology, cross-disciplinary cooperation among nursing science, computer science, and medical informatics has propelled technological innovation and application in this field (48). This interdisciplinary collaboration not only enhances AI’s potential in nursing management but also increases research diversity and global impact. For instance, journals like Cin-Computers Informatics Nursing (49) and Computational Intelligence and Neuroscience (49) bridge nursing and informatics, facilitating widespread technology dissemination. Furthermore, journals like the Journal of Medical Internet Research (50) and the Journal of Advanced Nursing (51) demonstrate how interdisciplinary collaboration drives innovation in smart nursing and personalized medicine. Consequently, such collaboration not only accelerates the application of AI technologies but also advances global nursing research and enhances overall care quality.

The article titled “Artificial Intelligence-based technologies in nursing: A scoping literature review of the evidence” (15) has become the most cited reference in its field due to its high relevance to current healthcare research trends and practical requirements. This study systematically addresses the application of AI in nursing, filling a critical gap in the literature where nursing practice is often overlooked within broader healthcare AI research. adopting a scoping review methodology, it provides a comprehensive overview of developmental stages, research quality, and implementation gaps, offering valuable reference points for future studies. Crucially, it not only summarizes the current research landscape but also identifies key shortcomings—such as limited clinical application, insufficient nurse involvement, and lack of ethical considerations—while proposing clear directions for improvement. These insights provide both theoretical foundations and practical guidance, making the study highly relevant to interdisciplinary researchers, practitioners, and policymakers. Furthermore, this article was published in the International Journal of Nursing Studies, one of the most prestigious and influential journals in the nursing field, recognized for its rigorous peer-review process and high impact factor (52). Its global readership and strong academic reputation significantly enhance the article’s visibility and credibility, further contributing to its high citation rate. The results of the co-cited reference analysis indicate that high-frequency publications primarily focus on the transformative role of AI in nursing management practice, methodological standardization, and clinical application situations. The most frequently co-cited reference is “How Artificial Intelligence is Changing Nursing” (53), highlighting the centrality of practice-oriented research in this field. It is followed by “Artificial Intelligence in Nursing: Priorities and Opportunities from an International Invitational Think-Tank” (27), which high co-cited frequency reflects its significant influence in setting academic agendas and policy directions. Additionally, methodological literature such as PRISMA-ScR (54), COREQ (55), and Scoping Review frameworks (56) also rank among the top 10, indicating the field’s strong emphasis on systematic and standardized research. Overall, the research on AI applications in nursing management is transitioning from an early exploratory phase toward greater structure and systematization. This knowledge evolution follows a trajectory characterized by practice-driven orientation, methodological rigor, and interdisciplinary integration.

The co-occurrence of keywords systematically reveals the thematic structure and development trends of AI research in nursing management. First, keywords like “AI,” “nursing management,” and “machine learning” emphasize the foundational focus on AI technology integration and clinical decision support. This is followed by keywords such as “nurse management,” “empowerment,” and “qualitative research,” which address managerial and behavioral perspectives in nursing, signaling the growing interest in leadership and organizational readiness for AI adoption. This finding aligns with the research by Özkaya & Körükcü (57), which indicates that “AI” has been the most frequently used keyword in nursing research in recent years, suggesting that research focus is shifting toward practical clinical applications and management practices. Next, the presence of terms like “data mining technology,” “big data,” and “information management” point to the critical role of data infrastructure and analytics in supporting AI applications. The inclusion of terms like “survey,” “questionnaire,” and “psychology” reveals an emphasis on psychosocial factors and the human side of AI adoption, particularly the perceptions and attitudes of nursing staff. Finally, keywords such as “heart failure,” “prediction model,” and “critical care” extend into the realm of clinical disease prediction and AI modeling, highlighting the application of AI in disease management and patient care. This structure illustrates a clear progression from technological advancements and data management systems to the psychosocial adaptation of AI technologies in nursing and ultimately to clinical applications in patient care. Such a development trajectory aligns with broader trends observed in the literature, where research has increasingly evolved from foundational educational and technical aspects to focus on practical AI applications, human factors, and interdisciplinary integration. Chang et al. (14) similarly observed in their bibliometric analysis of nursing AI trends that research focus has expanded from foundational education to medical record management and intelligent decision support, revealing a pronounced trend toward cross-domain integration. Keywords burst analysis further enhances the temporal dimension of this trend. Early research (1990–2009), on the basis of “nursing education” and “methodology,” established the foundational research framework for education and methodological standards. The mid-period, represented by “clinical practice” and “cohort study,” marked the transition into clinical trials and evidence-based application. In recent years, keywords like “deep learning,” “algorithm,” and “nursing administration” have rapidly emerged, signaling the deep integration of technology application with management and governance levels. Monaco et al. (58) also proposed a similar perspective in their macro-level review of nursing AI research from 2000 to 2024, suggesting that recent studies increasingly emphasize the dual pathways of “technological feasibility” and “interdisciplinary collaboration.”

Overall, this bibliometric study highlights the transformative trajectory of AI in nursing management—from exploratory innovation to applied practice, and from isolated development to global collaboration. Despite notable achievements, the field still faces structural challenges, including geographical disparities, uneven academic influence, and limited cross-disciplinary integration in certain regions. Among these, the fragmentation of institutional cooperation stands out as a critical barrier to the field’s advancement (59).

This fragmentation stems from several interrelated factors. First, the absence of a unified global or regional coordination framework often results in institutions prioritizing individual goals over collective strategies (60). Second, disciplinary silos between nursing management, artificial intelligence, and health informatics hinder collaboration due to differences in priorities, methodologies, and terminologies (6). Third, resource constraints—especially in low-income countries—limit participation in global research, as institutions may lack the funding, technical capacity, or workforce to engage in collaborative projects. The competitive nature of research funding further incentivizes siloed efforts over joint initiatives (13). Cultural differences, regulatory disparities, and geopolitical tensions also impede international cooperation (59). Divergent national policies on data privacy, AI ethics, and healthcare regulation complicate cross-border data sharing and slow the adoption of AI innovations in nursing across contexts (60). Additionally, language barriers and a lack of harmonized research standards exacerbate the difficulty of multinational collaboration, particularly in large-scale empirical studies (61).

This fragmentation can impede the flow of knowledge and best practices across countries, leading to duplication of efforts and reduced efficiency in scientific progress. It also constrains the development of globally interoperable AI systems, as solutions tend to reflect localized needs, datasets, and regulatory environments. Furthermore, researchers in low- and middle-income countries may face barriers to accessing high-impact collaboration networks, which can perpetuate global disparities in AI integration and nursing innovation.

To address these challenges, future policies should prioritize the development of international collaborative networks—particularly for applied AI research in nursing contexts (13). Transnational funding mechanisms that support multi-institutional projects can help overcome resource disparities and promote equitable participation across regions. Establishing standardized ethical and regulatory frameworks will further facilitate cross-border data interoperability while ensuring privacy protection and compliance with local regulations. Promoting interdisciplinary education—especially through curricula that integrate nursing science with artificial intelligence and data science—can cultivate a new generation of professionals equipped to lead cross-cultural, interdisciplinary collaboration in AI-enabled care management.

At the same time, the ethical dimensions of AI implementation must be addressed alongside these structural reforms. Algorithmic transparency, fairness, and data governance are critical considerations, particularly as AI systems increasingly influence clinical decision-making and administrative resource allocation. Without proper oversight, AI tools risk reinforcing systemic biases or compromising patient trust. Therefore, fostering algorithmic literacy and ethical awareness among healthcare professionals and decision-makers is essential. Together, these educational, regulatory, and collaborative efforts will ensure that AI in nursing management evolves not only as a technical innovation but also as a human-centered, ethically grounded advancement.

## 5 Limitations

Several limitations of this study should be acknowledged. First, the database was extracted solely from the WOSCO and Scopus. While they are authoritative academic sources, they may not fully capture relevant research indexed in other databases such as PubMed, Embase, or regional repositories, potentially affecting data comprehensiveness. Second, the analysis is confined to English-language publications, potentially leading to underrepresentation of research output from non-English-speaking regions and thus limiting the global scope of insights. Third, by its nature, bibliometric analysis focuses on publication and citation metrics without evaluating the quality, methodological rigor, or clinical applicability of the individual studies reviewed. Fourth, AI developments in nursing management evolve rapidly. The citation metrics and keyword bursts analyzed in this study reflect trends current as of August 2025 but may lag behind the latest advancements and emerging technologies. Finally, variations in AI application contexts across countries and regions, such as institutional infrastructure, nursing practices, healthcare policies, and technological maturity, may impact the generalizability of the findings.

## 6 Conclusion

This bibliometric study provides an overview of the development trajectory and knowledge landscape of AI in nursing management. Over the past decade, the field has entered a phase of rapid advancement, characterized by rising publication volumes, expanding global participation, and a trend toward broader integration of AI technologies into nursing workflows. Close collaboration has emerged among certain countries, and a knowledge ecosystem centered around a few prominent journals and institutions has taken form. However, despite these advances, the field remains somewhat fragmented, with limited collaboration at both the institutional and author levels, indicating a demand for stronger academic networks. Furthermore, frequent citations of methodological standards such as PRISMA-ScR and COREQ suggest growing awareness of research rigor and transparency. Finally, research hotspots have shifted from early focuses on nursing education and foundational methodologies toward current interests in deep learning, algorithmic applications, and AI-assisted clinical decision-making. This trend reflects the field’s progression from conceptual exploration to practical implementation.

Looking ahead, promoting AI-enabled nursing tools, strengthening ethical governance, and integrating AI into strategic leadership and policy-making will be crucial. Achieving this will require enhanced international and interdisciplinary collaboration—particularly between computer science, nursing informatics, and clinical practice. Ultimately, AI holds the potential to transform nursing management by enabling more efficient operations, data-driven decision-making, and personalized, patient-centered care.

## Data Availability

The original contributions presented in the study are included in the article/Supplementary material, further inquiries can be directed to the corresponding author.
