# Supplementary material for: The research hotspots and trends of artificial intelligence technology in nursing management: a bibliometric study
Source: Front Med (Lausanne). 2025 Dec 10;12:1710269. doi: 10.3389/fmed.2025.1710269 (PMC12727640; doi:10.3389/fmed.2025.1710269)
Supplement: Supplementary file 1 [file Data_Sheet_1.docx]

| NO. | Title | Journal | First author | Year |
| --- | --- | --- | --- | --- |
| 1 | Learning from management videos: the producers’ point of view | Nurse Educator | Gillies, Dee ann | 1990 |
| 2 | Depo Provera. Position paper on clinical use, effectiveness and side effects | The British Journal of Family Planning | Bigrigg, A | 1999 |
| 3 | Effect of the Growth Regulator Uniconazole on Biomass Allocation of Bareroot Loblolly Pine Seedlings | Southern Journal of Applied Forestry | Barnes, AD | 2004 |
| 4 | Autonomic dysreflexia and telehealth | The Canadian Nurse | Lapierre, NM | 2006 |
| 5 | Quality assessment and cataloguing of telemedicine applications | Journal of Telemedicine and Telecare | Bedini, R | 2006 |
| 6 | The use of telecommunications in teaching | Africa Journal of Nursing and Midwifery | Mcinerney, PA | 2007 |
| 7 | Staff members' perceptions of a ICT support package in dementia care during the process of implementation | Journal of nursing management | Engstrom, Maria | 2009 |
| 8 | Managing medical advice seeking in calls to Child Health Line | Sociology of Health & Illness | Butler, Carly W. | 2009 |
| 9 | Integrating telehealth in nursing curricula: can you hear me now? | Online Journal of Nursing Informatics | Gallagher-lepak, Susan | 2009 |
| 10 | Using videoconferencing to discuss themes of nursing management in university hospitals | ACTA Paulista de Enfermagem | Salvador, ME | 2010 |
| 11 | Jim Cato, EdD, RN, CRNA, CPEHR | Nurse Leader | Shaffer, Franklin a | 2010 |
| 12 | Postoperative care for the robotic surgery bowel resection patient | Gastroenterology Nursing | Brenner, Zara r | 2011 |
| 13 | Reducing cardiovascular disease risk in medically underserved urban and rural communities | American Heart Journal | Bove, Alfred A. | 2011 |
| 14 | Nurse adoption of continuous patient monitoring on acute post-surgical units: Managing technology implementation | Journal of Nursing Management | Jeskey, Mary | 2011 |
| 15 | Achieving the 'perfect handoff' in patient transfers: Building teamwork and trust | Journal of Nursing Management | Clarke, Diana | 2012 |
| 16 | Measuring tele-ICU impact: does it optimize quality outcomes for the critically ill patient? | Journal of Nursing Management | Goran, Susan F. | 2012 |
| 17 | Tailored Case Management for Diabetes and Hypertension (TEACH-DM) in a community population: Study design and baseline sample characteristics | Contemporary Clinical Trials | Crowley, Matthew J. | 2013 |
| 18 | E-health and care organization: Structuring of anti-cancer treatment in Franche-Comté | European Research in Telemedicine | Perrin, S | 2013 |
| 19 | The evaluation of the compatibility of electronic patient record (EPR) system with nurses' management needs in a developing country | International Journal of Medical Informatics | Kahouei, Mehdi | 2015 |
| 20 | Usability and feasibility of a tablet-based Decision-Support and Integrated Record-keeping (DESIRE) tool in the nurse management of hypertension in rural western Kenya | International Journal of Medical Informatics | Vedanthan, Rajesh | 2015 |
| 21 | Proposing a new conceptual model and an exemplar measure using health information technology to examine the impact of relational nurse continuity on hospital-acquired pressure ulcers | Advances in Nursing Science | Stifter, Janet | 2015 |
| 22 | Nursing Management Minimum Data Set: Cost-Effective Tool To Demonstrate the Value of Nurse Staffing in the Big Data Science Era | Nursing Economics | Pruinelli, Lisiane | 2016 |
| 23 | Promoting mHealth in Nursing Practice in China | Nursing Informatics 2016: Ehealth For All: Every Level Collaboration - From Project to Realization | Liu, Yisi | 2016 |
| 24 | Embedding robotic surgery into routine practice and impacts on communication and decision making: a review of the experience of surgical teams | Cognition, Technology and Work | Randell, Rebecca | 2016 |
| 25 | Research on Construction of Nursing Knowledge Portal Based on Big Data | 2017 6TH International Conference of Educational Innovation Through Technology (EITT) | Liu, Ying | 2017 |
| 26 | Bring Your Own Device and Nurse Managers' Decision Making | Cin-computers Informatics Nursing | Martinez, Karen | 2017 |
| 27 | “Leading Better Care”: An evaluation of an accelerated coaching intervention for clinical nursing leadership development | Journal of Nursing Management | Cable, Stuart | 2018 |
| 28 | Pharmacy-based hypertension care employing mHealth in Lagos, Nigeria - a mixed methods feasibility study | BMC Health Services Research | Nelissen, Heleen E. | 2018 |
| 29 | Giving advice to callers with mental illness: adaptation among telenurses at Swedish Healthcare Direct | International Journal of Qualitative Studies on Health and Well-being | Bjoerkman, Annica | 2019 |
| 30 | Nursing Value User Stories A Value Measurement Method for Linking Nurse Contribution to Patient Outcomes | Cin-computers Informatics Nursing | Moon, Lisa A. | 2019 |
| 31 | Nurses' Readiness in the Adoption of Hospital Electronic Information Management Systems in Ghana: The Application of the Structural Equation Modeling and the UTAUT Model | Sage Open | Zhou, Lulin | 2020 |
| 32 | Precision health: A nursing perspective | International Journal of Nursing Sciences | Fu, Mei R. | 2020 |
| 33 | Research on nursing management based on big data | Proceedings - 2020 12th International Conference on Measuring Technology and Mechatronics Automation, ICMTMA 2020 | Zan, Tao | 2020 |
| 34 | High-Fidelity Simulation in an Undergraduate Ambulatory Care Nursing Course | Nursing Education Perspectives | Coburn, Caroline, V | 2020 |
| 35 | District nurses views on and experiences with a telemedicine educational programme in palliative care | Scandinavian Journal of Caring Sciences | Bauer, Eithne hayes | 2020 |
| 36 | Patient willingness to undergo a two-week free trial of a telemedicine service for coronary artery disease after coronary intervention: A mixed-methods study | Journal of Nursing Management | Lin, Yueh-Hsiu | 2020 |
| 37 | A scoping review of the nursing workforce's changing demography: Supporting Baby-Boomer nurses | Journal of Nursing Management | Gan, Ivan | 2020 |
| 38 | Translating Data From an Electronic Prescribing and Medicines Administration System Into Knowledge Application to Doctor-Nurse Time Discrepancy in Antibiotic Ordering and Administration | Medical Care | Van Wilder, Astrid | 2020 |
| 39 | Robot-Assisted Total Pancreatectomy With Autologous Islet Cell | Aorn Journal | Williams, Germaine M. | 2020 |
| 40 | Implementing national electronic health records in nursing homes in Tyrol: A nursing management perspective | Studies in Health Technology and Informatics | Schaller, Michael | 2020 |
| 41 | Predicted Influences of Artificial Intelligence on the Domains of Nursing: Scoping Review | JMIR Nursing | Buchanan, Christine | 2020 |
| 42 | The role of structural empowerment in predicting computer use among Jordanian nurses: A cross-sectional study | Journal of Nursing Management | Ta'an, Wafa'a f | 2021 |
| 43 | Optimization of Clinical Nursing Management System Based on Data Mining | COMPLEXITY | Chen, Yongxia | 2021 |
| 44 | Effects of mHealth-based interventions on health literacy and related factors: A systematic review | Journal of Nursing Management | Lin, Yueh-hsiu | 2021 |
| 45 | Medical Internet of Things to Realize Elderly Stroke Prevention and Nursing Management | Journal of Healthcare Engineering | Li, Xin | 2021 |
| 46 | Intention to migrate among the next generation of Turkish nurses and drivers of migration | Journal of Nursing Management | Oncu, Emine | 2021 |
| 47 | Analytics and Lean Health Care to Address Nurse Care Management Challenges for Inpatients in Emerging Economies | Journal of Nursing Scholarship | Moreno-Fergusson, Maria Elisa | 2021 |
| 48 | Machine learning-based patient classification system for adult patients in intensive care units: A cross-sectional study | Journal of Nursing Management | An, Ran | 2021 |
| 49 | Robotic Surgery and Nursing | Robotic Surgery and Nursing | Wang, Gongxian | 2021 |
| 50 | Human-centered implementation research: a new approach to develop and evaluate implementation strategies for strengthening referral networks for hypertension in western Kenya | BMC Health Services Research | Pillsbury, Mc Kinsey M. | 2021 |
| 51 | Application and Effectiveness of Big Data and Artificial Intelligence in the Construction of Nursing Sensitivity Quality Indicators | Journal of Healthcare Engineering | Chen, Aie | 2021 |
| 52 | Medical Information Mining-Based Visual Artificial Intelligence Emergency Nursing Management System | Journal of Healthcare Engineering | Dong, Aihua | 2021 |
| 53 | Emergency preparedness during the COVID-19 pandemic: Perceptions of oncology professionals and implications for nursing management from a qualitative study | Journal of Nursing Management | Marshall, Victoria k | 2021 |
| 54 | Association of artificial intelligence use and the retention of elderly caregivers: A cross-sectional study based on empowerment theory | Journal of Nursing Management | Wang, Ying | 2022 |
| 55 | Artificial Intelligence -based technologies in nursing: A scoping literature review of the evidence | International Journal of Nursing Studies | von Gerich, Hanna | 2022 |
| 56 | Identification of elderly patients at risk for 30-day readmission: Clinical insight beyond big data prediction | Journal of Nursing Management | Flaks-manov, Natalie | 2022 |
| 57 | Artificial Intelligence Technology-Based Medical Information Processing and Emergency First Aid Nursing Management | Computational and Mathematical Methods in Medicine | Liu, Qing | 2022 |
| 58 | Artificial intelligence and robot nurses: From nurse managers' perspective: A descriptive cross-sectional study | Journal of Nursing Management | Ergin, Eda | 2022 |
| 59 | Construction of Nursing Quality Evaluation Index System Based on Big Data Assisted Analysis in the Context of Intelligent Medical Treatment | Mobile Information Systems | Wang, WanLi | 2022 |
| 60 | Ethical issues of smart home-based elderly care: A scoping review | Journal of Nursing Management | Zhu, Junhong | 2022 |
| 61 | Management Strategy of Alzheimer's Patients under the Medical-Care Integration Model Based on Big Data Evaluation | Biomed Research International | Sun, Wei | 2022 |
| 62 | Effect of AI deep learning techniques on possible complications and clinical nursing quality of patients with coronary heart disease | Food Science and Technology | Zhang, Pengbo | 2022 |
| 63 | Implementation of Hospital-to-Home Model for Nutritional Nursing Management of Patients with Chronic Kidney Disease Using Artificial Intelligence Algorithm Combined with CT Internet | Contrast Media & Molecular Imaging | Chen, Xing | 2022 |
| 64 | The role of artificial intelligence in enhancing clinical nursing care: A scoping review | Journal of Nursing Management | Ng, Zi qi pamela | 2022 |
| 65 | Trends in artificial intelligence in nursing: Impacts on nursing management | Journal of Nursing Management | Chang, Ching-Yi | 2022 |
| 66 | Nurse leaders' and digital service developers' perceptions of the future role of artificial intelligence in specialized medical care: An interview study | Journal of Nursing Management | Laukka, Elina | 2022 |
| 67 | Critical role of information and communication technology in nursing during the COVID-19 pandemic: A qualitative study | Journal of Nursing Management | Yoo, Hye Jin | 2022 |
| 68 | In-field pine seedling counting using end-to-end deep learning for inventory management | 2022 ASABE Annual International Meeting | Puhl, Rafael bidese | 2022 |
| 69 | Adoption of novel biomarker test parameters with machine learning-based algorithms for the early detection of sepsis in hospital practice | Journal of Nursing Management | Manetti, Stefania | 2022 |
| 70 | Cancer patients and telenursing interventions in Italy: a systematic review | World Cancer Research Journal | de Leo, A | 2022 |
| 71 | Identification of telehealth nursing approaches in the light of the COVID-19 pandemic-A literature review | Journal of Nursing Management | Burilova, Petra | 2022 |
| 72 | Leaders’ innovation expectation and nurses’ innovation behaviour in conjunction with artificial intelligence: The chain mediation of job control and creative self-efficacy | Journal of Nursing Management | Li, Xianmiao | 2022 |
| 73 | Application of the Improved Clustering Algorithm in Operating Room Nursing Recommendation under the Background of Medical Big Data | Journal of Healthcare Engineering | Wu, Xiaofang | 2022 |
| 74 | Development of a predictive inpatient falls risk model using machine learning | Journal of Nursing Management | Ladios-Martin, Mireia | 2022 |
| 75 | Artificial intelligence based prediction models for individuals at risk of multiple diabetic complications: A systematic review of the literature | Journal of Nursing Management | Gosak, Lucija | 2022 |
| 76 | How robots impact nurses' time pressure and turnover intention: A two-wave study | Journal of Nursing Management | Huang, Tzu-ling | 2022 |
| 77 | Experiences and perceptions of final-year nursing students of using a chatbot in a simulated emergency situation: A qualitative study | Journal of Nursing Management | Rodriguez-arrastia, Miguel | 2022 |
| 78 | Research Trends in Artificial Intelligence-Associated Nursing Activities Based on a Review of Academic Studies Published From 2001 to 2020 | CIN-COMPUTERS INFORMATICS NURSING | Hwang, Gwo-Jen | 2022 |
| 79 | The prevalence of stress-related outcomes and occupational well-being among emergency nurses in the Netherlands and the role of job factors: A regression tree analysis | Journal of Nursing Management | de Wijn, Anne Nathal | 2022 |
| 80 | The Construction and Effect Analysis of Nursing Safety Quality Management Based on Data Mining | Computational Intelligence and Neuroscience | Yang, Yimei | 2022 |
| 81 | Early Nurse Management Experiences from Finnish COVID-19 Hubs: An In-Action Review | International Journal of Environmental Research and Public Health | Nevala, Mari s | 2022 |
| 82 | Evolving with technology: Machine learning as an opportunity for operating room nurses to improve surgical care-A commentary | Journal of Nursing Management | Irani, Cameron S. S. | 2022 |
| 83 | Artificial intelligence for falls management in older adult care: A scoping review of nurses’ role | Journal of Nursing Management | O'connor, Siobhan | 2022 |
| 84 | Health and social care frontline leaders' perceptions of competence management in telemedicine in Finland: An interview study | Journal of Nursing Management | Myllymäki, Suvi | 2022 |
| 85 | A PDCA Model for Disinfection Supply Rooms in the Context of Artificial Intelligence to Reduce the Incidence of Adverse Events and Improve the Disinfection Compliance Rate | Journal of Healthcare Engineering | Wang, Yunxia | 2022 |
| 86 | Artificial intelligence in health care: Implications for nurse managers | Journal of Nursing Management | Peltonen, Laura-Maria | 2022 |
| 87 | The changing role of patients, and nursing and medical professionals as a result of digitalization of health and heart failure care | Journal of Nursing Management | Boyne, Josiane J. | 2022 |
| 88 | Is there a gap between artificial intelligence applications and priorities in health care and nursing management? | Journal of Nursing Management | Chen, Yanjiao | 2022 |
| 89 | Identifying nursing sensitive indicators from electronic health records in acute cardiac care? Towards intelligent automated assessment of care quality | Journal of Nursing Management | von Gerich, Hanna | 2022 |
| 90 | Artificial intelligence-based intelligent surveillance for reducing nurses’ working hours in nurse–patient interaction: A two-wave study | Journal of Nursing Management | Huang, Kai | 2022 |
| 91 | Construction and Implementation of Procedural Nursing System for General Surgery Laparoscopic Surgery Based on Deep Learning | Journal of Healthcare Engineering | Zheng, Yonghong | 2022 |
| 92 | Application of Data Mining Technology-Based Nursing Risk Management in Emergency Department Care | Mathematical Problems in Engineering | Han, Weiwei | 2022 |
| 93 | Management technology for implementing the Systematization of Nursing Care | REVISTA DA ESCOLA DE ENFERMAGEM DA USP | de Sousa, Anderson Reis | 2022 |
| 94 | Leadership in the context of digital health services: A concept analysis | Journal of Nursing Management | Laukka, Elina | 2022 |
| 95 | A Clinical Nursing Management System and Method Based on Data Mining Algorithm | Lecture Notes on Data Engineering and Communications Technologies | Yang, Lixia | 2022 |
| 96 | Medical Intelligent System and Orthopedic Clinical Nursing Based on Graph Partition Sampling Algorithm | Computational Intelligence and Neuroscience | Heng, Xiucheng | 2022 |
| 97 | On the Way to the Future—Assistant Robots in Hospitals and Care Facilities | Lecture Notes in Computer Science | Radic, Marija | 2022 |
| 98 | Factors affecting the length of stay in the emergency department for critically Ill patients transferred to regional emergency medical center | Nursing Open | Lee, Hyungbok | 2023 |
| 99 | Application of a new nano-TiO2 composite antibacterial agent in nursing management of operating room: Based on real-time information push assistant system | Preventive Medicine | Xiaoyan, An | 2023 |
| 100 | The rationale and guiding principles to design a psychiatric curriculum for primary care nurses of India | Journal of Family Medicine and Primary Care | Ohri, Uma | 2023 |
| 101 | In what ways are HR analytics and artificial intelligence transforming the healthcare sector? | ASIA Pacific Journal of Human Resources | Cavanagh, Jillian | 2023 |
| 102 | Incidence and predictors of abdominal pain after transarterial chemoembolization of hepatocellular carcinoma: a single-center retrospective study | European Journal of Oncology Nursing | Du, Qian-qian | 2023 |
| 103 | Hospital Performance Management: Implementation of Real-Time Monitoring System for Clinical Sector | Data-Centric AI Solutions and Emerging Technologies in the Healthcare Ecosystem | Jadhav, Babasaheb | 2023 |
| 104 | A comparison between Pixel-based deep learning and Object-based image analysis (OBIA) for individual detection of cabbage plants based on UAV Visible-light images | Computers and Electronics in Agriculture | Ye, Zhangxi | 2023 |
| 105 | Self-Care Behavior and Associated Factors of Nursing Students with Dysmenorrhea: A Structural Equation Model | Journal of Nursing Management | Chen, Jinpei | 2023 |
| 106 | The application of chatbot in gastroenterology nursing | Gastroenterology and Endoscopy | Zhao, Yang | 2023 |
| 107 | Implementation and Outcomes of an Advanced Nurse Practitioner Telephone Chest Pain Clinic Developed in Response to the COVID-19 Pandemic | The Journal For Nurse Practitioners | Ingram, Shirley | 2023 |
| 108 | Nursing care management in substitutive renal therapy in patients with COVID-19: integrative review | Enfermeria Nefrologica | Carvalho-De Alencar, Cícero damon | 2023 |
| 109 | Perception of nurses on the use of mobile phone text messaging for the management of diabetes mellitus in rural Ghana | Nursing Open | Abdulai, Eliasu | 2023 |
| 110 | Applications of Artificial Intelligence in Nursing Care: A Systematic Review | Journal of Nursing Management | Martinez-ortigosa, Adrian | 2023 |
| 111 | Tracheal rupture treated with robotic surgery: the importance of evidence-based practice in nursing management. A case report | Assistenza Infermieristica e Ricerza | Migliaccio, Stefano | 2023 |
| 112 | Prediction of Nursing Need Proxies Using Vital Signs and Biomarkers Data: Application of Deep Learning Models | Journal of Clinical Nursing | Baek, Yunmi | 2024 |
| 113 | Design of Nursing Information Collection System Based on Artificial Intelligence Big Data Analysis Algorithm | ACM International Conference Proceeding Series | Tang, Anyu | 2024 |
| 114 | Big data research in nursing: A bibliometric exploration of themes and publications | Journal of Nursing Scholarship | Li, Bo | 2024 |
| 115 | Big Data Analysis and Quality Control of Assisted Reproductive Technology Based on Association Rule Mining | Proceedings - 2024 Asia-Pacific Conference on Software Engineering, Social Network Analysis and Intelligent Computing, SSAIC 2024 | Li, Bing | 2024 |
| 116 | The importance of standardization in nursery management and production: a blockchain approach | Acta Horticulturae | Sortino, A | 2024 |
| 117 | Effects of Job Crafting and Leisure Crafting on Nurses' Burnout: A Machine Learning-Based Prediction Analysis | Journal of Nursing Management | Guo, Yu-fang | 2024 |
| 118 | Impact of Artificial Intelligence-Based Technology on Nurse Management: A Systematic Review | Journal of Nursing Management | Gonzalez-Garcia, Alberto | 2024 |
| 119 | Telemedicine and Point-of-Care Devices: A Tale of Success for the Nurses' Management of Patients with Chronic Illness | Cardiology (Switzerland) | Marziliano, Nicola | 2024 |
| 120 | Development of a supportive care framework for breast cancer survivor's unmet needs: A modified Delphi study | Journal of Clinical Nursing | Dai, Qian | 2024 |
| 121 | Predicting nursing workload in digestive wards based on machine learning: A prospective study | BMC Nursing | Song, Yulei | 2024 |
| 122 | Patient satisfaction analysis of robot-assisted minimally invasive adrenalectomy: a single-center retrospective study | Journal of Robotic Surgery | Xue, Yao | 2024 |
| 123 | The Utilization of Natural Language Processing for Analyzing Social Media Data in Nursing Research: A Scoping Review | Journal of Nursing Management | Wang, Zhenrong | 2024 |
| 124 | The Impact of Medical Explainable Artificial Intelligence on Nurses' Innovation Behaviour: A Structural Equation Modelling Approach | Journal of Nursing Management | Li, Xianmiao | 2024 |
| 125 | Nursing Students' Personality Traits and Their Attitude toward Artificial Intelligence: A Multicenter Cross-Sectional Study | Journal of Nursing Management | Salem, Gihan Mohamed Mohamed | 2024 |
| 126 | Mobile Application-Based Interventions for People with Heart Failure: A Systematic Review and Meta-Analysis | Journal of Nursing Management | Ni, Yun-xia | 2024 |
| 127 | Predicting New Graduate Nurses' Retention during Transition Using Decision Tree Methods: A Longitudinal Study | Journal of Nursing Management | Lee, Taewha | 2024 |
| 128 | Efficacy of a Standardized Process in Optimizing Appropriate Use of Progressive Care Unit Beds in a Tertiary Care Facility | Dimensions of Critical Care Nursing | Stempek, Susan B. | 2024 |
| 129 | Perioperative Nursing Considerations for Transurethral Resection Prostatectomy | Journal of the Medical Association of Thailand | Ratanatherawichian, Yananan | 2024 |
| 130 | Artificial Intelligence in the Organization of Nursing Care: A Scoping Review | Nursing Reports | Ventura-Silva, Joao | 2024 |
| 131 | Assessing Visitor Expectations of AI Nursing Robots in Hospital Settings: Cross-Sectional Study Using the Kano Model | JMIR Nursing | Kang, Aimei | 2024 |
| 132 | Patterns and Trends in Global Nursing Robotics Research: A Bibliometric Study | Journal of Nursing Management | Zhang, Shan | 2025 |
| 133 | Transformative Insights into Community-Acquired Pressure Injuries Among the Elderly: A Big Data Analysis | Healthcare | Shafran-Tikva, Sigal | 2025 |
| 134 | Establishment and validation of a prediction model for compassion fatigue in nursing students | BMC Nursing | Zhang, Huiling | 2025 |
| 135 | Ethical Artificial Intelligence in Nursing Workforce Management and Policymaking: Bridging Philosophy and Practice | Journal of nursing management | Park, Claire Su-Yeon | 2025 |
| 136 | Postoperative feedback-oriented health education in ambulatory robot-assisted adrenalectomy: a single-center nursing intervention cohort study | Journal of Robotic Surgery | Yao, Xue | 2025 |
| 137 | Artificial Intelligence in Nursing Decision-Making: A Bibliometric Analysis of Trends and Impacts | Nursing Reports | Hu, Mengdie | 2025 |
| 138 | Progress in lung cancer study coupled with cognitive frailty in elderly individuals | Geriatric Nursing | Liu, Chenli | 2025 |
| 139 | Navigating Society 5.0: Integrating Strength-Based Leadership and Job Crafting for Improved Nursing Outcomes | Journal of Nursing Management | Wang, Xule | 2025 |
| 140 | Tech-Enhanced Forest Nursery Management: Harnessing Embedded Systems for Plant Health Monitoring and Growth Forecasting | IEEE Internet of Things Journal | Ranjan Jena, Manas | 2025 |
| 141 | Artificial Intelligence and Nursing Management: Opportunities, Challenges, and Ethical Considerations—A Scoping Review | Journal of Nursing Management | Katebi, Maryam | 2025 |
| 142 | Artificial intelligence (AI) in nursing administration: Challenges and opportunities | PLOS ONE | Qaladi, Omar | 2025 |
| 143 | Perspective on the nursing management for gestational diabetes mellitus: A perspective | Medicine (United States) | Fan, Ya-ting | 2025 |
| 144 | Effects of viewing angle and field of view on detection, tracking, and counting of pine seedlings towards automated forest nursery inventory | Smart Agricultural Technology | Mulaka, Ashish reddy | 2025 |
| 145 | Implementation and clinical management status of smart care and smart technology systems in Shandong Province, China: a multicentre cross-sectional study | Health and Technology | Wu, Yu | 2025 |
| 146 | Factors of Nurses Using Mobile Applications to Provide Home Nursing Care: A Social Cognitive Theory Perspective | Journal of Nursing Management | Cheng, Jing | 2025 |
| 147 | Sensor Based Environmental Control System for Efficient Nursery Management | 2025 International Conference on Electrical, Computer and Communication Engineering, ECCE 2025 | Mortuza, Mdgolam | 2025 |
| 148 | Nursing management of tube feeding among geriatric patients in long-term care facilities: Summary of best evidence-practiced strategies | Geriatric Nursing | Fu, Manyi | 2025 |
| 149 | Nursing Robots Can Reduce Nursing Workload in General Adult Wards: A Two-Phase Study | Journal of Nursing Management | Song, Yulei | 2025 |
| 150 | Strategies utilized and challenges faced by stakeholders while managing epilepsy in African schools: A scoping review | Epliepsy & Behavior | Dzah, Seth Selassie | 2025 |
| 151 | Immersive virtual reality simulation versus screen-based virtual simulation: An examination of learning outcomes in nursing education | Clinical Simulation in Nursing | Dong, Cindy | 2025 |
